# From orientation to integration: organizational leadership and mentorship for newly graduated nurses – a qualitative hermeneutic-phenomenological study

**DOI:** 10.1186/s12912-026-04886-4

**Published:** 2026-06-15

**Authors:** Jette Henriksen, Ingrid Villadsen Kristensen

**Affiliations:** 1 Faculty of Nursing and Health Sciences, Nord University, Levanger, Norway; 2 Department of Medicine, Gødstrup Hospital, Gødstrup, Denmark; 3 VIA Nursing Holstebro, VIA University College, Holstebro, Denmark

**Keywords:** Onboarding, Newly graduated nurses, Mentorship, Leadership, Professional identity, Qualitative research, Transition to practice

## Abstract

**Background:**

The transition from student to registered nurse is a demanding period marked by high expectations, emotional strain, and a need for rapid competence development. Structured onboarding programs are increasingly used to support newly graduated nurses, yet little is known about how mentors and leaders perceive their significance. This study explores these perspectives to deepen understanding of organizational and relational aspects of onboarding.

**Methods:**

A qualitative hermeneutic‑phenomenological design informed by Paul Ricoeur’s theory of interpretation was applied. Eight semi‑structured interviews with mentors and leaders from medical, surgical, and oncology departments were conducted. Data were analyzed through Ricoeur’s three interpretive phases: naïve reading, structural analysis, and critical interpretation.

**Findings:**

Four themes described how mentors and leaders understand and enact their roles in supporting newly graduated nurses. Balancing professional commitment in nursing onboarding highlighted the importance of early experiences for confidence and long‑term retention. Navigating individuality and structure in onboarding showed how standardized expectations are combined with tailored support. Being present yet invisible illustrated how mentors create psychological safety while promoting independence and reflective learning. Oscillating between standardized competence and evolving professional identity emphasized that the structured program strengthens clinical competence, supports identity formation, and contributes to a more stable workforce. There is an indication that such structured programs support care quality, competence development, professional identity, and retention.

**Conclusion:**

Mentors and leaders perceive structured onboarding as a relational and developmental process that extends far beyond administrative orientation. The findings suggest that structured programs enhance psychological safety, promote competence development, and foster a coherent professional identity. These elements contribute not only to newly graduated nurses’ successful transition but also to workforce stability and care quality. Investing in comprehensive onboarding initiatives is therefore essential for sustainable nursing practice.

**Supplementary Information:**

The online version contains supplementary material available at 10.1186/s12912-026-04886-4.

## Background

The transition from nursing student to fully qualified nurse is a critical period that requires careful planning and support from mentors and leaders [1, 2]. Newly graduated nurses often face a steep learning curve as they transition from academic settings to real-world clinical environments [1, 3, 4]. Leaders and mentors provide the necessary guidance and support, helping them navigate complex medical procedures, patient interactions, and hospital protocols [5, 6]. Leaders and mentors play a critical role in the professional development of newly graduated nurses [7]. They offer hands-on training, share valuable insights from their own experiences, and provide constructive feedback, which helps newly graduated nurses refine their clinical skills and build confidence in their abilities [7, 8]. To ensure a coherent transition there is a need of strategies and initiatives. These include mentoring programs where experienced nurses guide newly graduated ones, as well as structured introductory courses that allow newly graduated nurses to become familiar with workplace routines and procedures [8, 9]. Additionally, the leaders offer ongoing professional development and training, which helps the newly graduated nurses build their skills and confidence in their new role. By creating a supportive and learning-oriented environment, leaders actively work to reduce stress and promote a positive start to the career for the newly graduated nurses [10, 11].

Onboarding programs for newly graduated nurses play a crucial role in ensuring a successful transition from student to professional practice [6, 12]. These programs are designed to support the newly graduated nurses in their first time at the workplace and often include a combination of structured training, mentoring schemes, and ongoing professional development [13]. Several hospitals in Denmark have implemented introductory programs for new staff. The purpose of these initiatives is to increase the confidence and independence of the newly graduated nurses and strengthen their professional identity [14, 15].

The initial phase of a nursing career can be overwhelming and stressful [16, 17]. Research shows that structured onboarding programs can reduce stress and uncertainty among newly graduated nurses, which in turn can lead to higher job satisfaction and lower staff turnover [18, 19]. With an increasing need for nurses in the healthcare system, it is therefore essential to invest in effective onboarding programs that can retain the newly graduated nurses and ensure a high standard of patient care. Having supportive leaders and mentors can significantly impact on the emotional well-being of newly graduated nurses. They offer a safe space for discussing challenges, fears, and uncertainties, which can reduce anxiety and prevent burnout [20].

Newly graduated nurses experience onboarding programs as an important support in their transition from student to full-time professionals. Structured onboarding programs help them feel more confident and competent in their new roles [21, 22].

Furthermore, newly graduated nurses highlight that the onboarding programs contribute to a better understanding of workplace routines and procedures, which reduces stress and uncertainty [19] Leaders and mentors facilitate the integration of newly graduated nurses into the healthcare team. Introduction programme helps newly graduated nurses understand the dynamics of the workplace, foster positive relationships with colleagues, and promote a collaborative work environment, which is crucial for effective patient care. This is especially important at a time when the healthcare system is experiencing increasing work pressure and a shortage of experienced nurses [13].

Effective mentorship and leadership are linked to higher job satisfaction and retention rates among newly graduated nurses. When newly graduated nurses feel supported and valued, they are more likely to stay in their positions and pursue long-term careers in nursing, which benefits the healthcare system. Ultimately, the presence of skilled leaders and mentors ensures that newly graduated nurses are well-prepared to provide high-quality patient care. By fostering a culture of continuous learning and professional growth, leaders and mentors contribute to better patient outcomes and overall healthcare excellence [23, 24].

Overall, newly graduated nurses find that a well-organized onboarding program can make a big difference to their well-being and professional development, which in turn can lead to higher job satisfaction and lower staff turnover. But there is a lack of knowledge and insight into the significance of a structured introductions programme for newly graduated nurses from the perspective of the mentor and leaders. Therefore, the overall aim of this study is to provide insight into the significance of a structured introductions programme for newly graduated nurses from the perspective of the mentor and leader.

## Methods

### Design

The study is based on a hermeneutic-phenomenological approach with a qualitative research design, focusing on understanding and interpreting the participants’ experiences. Ricoeur’s combination of phenomenology and critical hermeneutic philosophy enables the attainment of understanding from a text and the achievement of new insights through critical interpretation [25, 26].

### Setting

The introduction programme for newly graduated nurses takes place in inpatient wards at a regional hospital in the Central Denmark Region. The programme consists of four modules and spans over two years. The introduction programme includes a general introduction to the hospital, training in the specific department, specialties, and clinic, as well as expectation meetings involving the head nurse and the introduction coordinator at the beginning and end of each module. Nurses in introduction positions have placements in other departments and sectors and participate in group supervision aimed at developing competencies related to patient care processes and the department’s overall care and treatment offerings [5]. The introduction coordinator is responsible for planning and coordinating the introduction programs. Furthermore, introduction coordinators have extensive clinical experience in medical specialty and pedagogical experience [5].

### Participants

A total of eight informants participated in the study. Three of them held positions as leaders for the newly graduated nurses, while the remaining five served as mentors responsible for supporting the professional development of the newly qualified staff. All informants had prior experience in utilizing the implemented introductory programs in clinical practice. Their experiences were therefore considered particularly valuable in exploring the perceived impact of these programs on individual professional development, collaborative work environments, and the overall quality of nursing within the department. The study participants represented departments within medicine, surgery, and oncology.

### Data collection

Semi-structured individual interviews were conducted based on open and dynamic interview questions to gain in-depth insight into how introduction coordinators and head nurses perceive their role in implementing a structured introduction program. The interview guide was developed specifically for the purposes of this study. An English version of the interview guide is provided in the supplementary file. Eight participants, who are either introduction mentors or head nurses, were interviewed. The number of participants was not decided by the principle of data saturation, as the richness of qualitative data does not necessarily increase with the number of participants or volume of text. Instead, throughout the data collection process, ongoing reflexive evaluation ensured that the material provided sufficient insight to address the aim [27]. The questions included, among others: How would you describe the impact the introduction programme has had on the workplace community in your department? In your view, how has the introduction programme influenced the quality of nursing in your department? How do you understand the significance of the introduction programme for newly graduated nurses?

Inspired by Ricoeur [26], the interviews allowed participants to share their experiences. The data collection process was flexible and tailored to each participant to ensure a comprehensive and nuanced understanding. This approach enabled the interviewer to interpret their experiences and their significance and ask follow-up questions, thereby enhancing the quality of the interviews [26, 28]. Hereby, the interviews will provide in-depth and personal insights into the participants’ experiences and perspectives regarding the implementation of structured introduction programs for newly graduated nurses. The first author collected data and was not employed in the department. The interviews were recorded and subsequently transcribed. The interviews lasted between 28 and 50 min and were conducted in 2023.

### Data analysis

The analysis was inspired by Paul Ricoeur’s three levels of interpretation. Initially, the researchers (authors) engaged in naive listening. This phase involved an open and preliminary interpretation, aimed at identifying the meaning of the narratives without engaging in deeper analysis.

Subsequently, a structural analysis was conducted to identify and examine the central themes and underlying structures within the data. This stage entailed a systematic and thorough interpretation of the material, allowing for a more nuanced understanding of its components.

Finally, an in-depth interpretation was carried out, wherein the researchers (authors) explored the previously identified themes and structures to uncover the deeper meanings and intentions embedded in the narratives. This phase required a critical and reflective approach, facilitating a comprehensive understanding of the data. An illustrative example of the structural analysis is presented in Table 1.

**Table 1:** Example of the structural analysis

Units of meaning What is said	Units of significance What the text talks about	Themes Emergence of key themes
“It is usually their first nursing job. If they get a good start, they might want to stay in the profession.” (R6).	Underscores how initial experiences shape not only competence but also long-term attachment to the profession.	Balancing professional commitment in nursing onboarding

### Ethical considerations

The study was reported to the Danish Data Protection Agency [29] to ensure compliance with national data protection regulations. According to Danish law this qualitative study does not require ethical approval [30]. The ethical principles of the Helsinki Declaration and the ethical guidelines for nursing research were followed [31, 32]. Participants received written information describing the purpose and method of the study, as well as how anonymity requirements are met. Additionally, participants received verbal information prior to each interview, and informed consent was obtained. The informants were anonymized, and data was stored on a secure research drive.

### Findings

The following findings section presents four themes derived from interviews with mentors and the unit leader involved in the onboarding of newly graduated nurses. These themes reflect how the participants understand and enact their roles in supporting newcomers, and together they illustrate the organizational, relational, and developmental dynamics that shape the onboarding process. The themes are: “Balancing professional commitment in nursing onboarding”, “Navigating individuality and structure in onboarding”, “Being present yet invisible: The paradox of mentorship, and “Oscillating between standardized competence and evolving professional identity”. In their entirety, they provide insight into how structured support, emotional safety, and professional expectations intersect to influence nurses’ early transition into clinical practice.

### Balancing professional commitment in nursing onboarding

One of the informants has served as the introduction coordinator since 2014, demonstrating a long-standing commitment to onboarding newly graduated nurses. She sees her role as both a mentor, role model, and facilitator, emphasizing the importance of a positive first experience in the profession.

It’s usually their first nursing job. If they get a good start, they might want to stay in the profession. (R6)

This statement highlights the crucial influence of early professional experiences on nurses’ long-term commitment to the profession. The transition from education to clinical practice is a formative period in which newly graduated nurses begin to build their professional identity, develop essential competencies, and assess whether they feel they belong in the clinical environment. When this initial phase is well-structured, supportive, and welcoming, it can strengthen nurses’ confidence, promote feelings of mastery, and reduce the stress often associated with entering the workforce. Such positive early experiences help reinforce motivation and self-efficacy, making it more likely that newly graduated nurses view nursing as a sustainable and meaningful career.

This indicates a supportive introduction also has broader organizational implications. Healthcare systems facing persistent challenges with turnover and workforce shortages increasingly recognize that the quality of onboarding plays a strategic role in retention. When newly graduated nurses experience guidance, recognition, and psychological safety, they are more inclined to remain in their positions and continue developing within the profession. In contrast, negative or fragmented introductory experiences such as insufficient support, lack of supervision, or overwhelming workload can contribute to early discouragement and increase the risk of leaving the profession altogether.

Thus, the introduction period should be understood not merely as an administrative process but as a critical intervention point that influences both individual well-being and the long-term stability of the nursing workforce. A positive start supports competence, confidence, and professional belonging, while simultaneously contributing to workforce retention in a field where sustaining qualified staff is an ongoing challenge.

### Navigating individuality and structure in onboarding

The introduction program is described as highly structured and intentional. The program is adapted to individual needs and backgrounds, ensuring that all new employees regardless of whether they are in an official introduction position receive support. The structure allows for both standardization and flexibility.

It’s very scheduled - what I’m supposed to do and when. (R4)

The quote illustrates a clear expectation-setting process within the program, where long-term competencies are defined in advance. This suggests a deliberate effort to balance individualized support with standardized outcomes, ensuring that all new employees regardless of their starting point are guided toward a shared professional benchmark over time. This approach also serves an important quality-assurance function, ensuring that developmental pathways are not left to individual discretion alone but instead follow a consistent pedagogical framework.

At the same time, predefined competency targets help balance individual variation with collective expectations. New employees enter the workplace with diverse backgrounds, strengths, and learning needs, yet a shared set of long-term outcomes ensures that everyone is ultimately working toward the same professional benchmark. This contributes to equity in training and reduces the risk of arbitrary or uneven development across individuals or departments. Moreover, having articulated goals enables mentors, supervisors, and coordinators to align their support, feedback, and assessment practices with the broader developmental trajectory of the programme.

Overall, such structured expectation-setting signals a proactive and intentional approach to professional growth. It allows organizations to shape learning in a strategic manner, emphasizing progression, continuity, and shared standards while still leaving room for individualized adaptation along the way.

### Being present yet invisible: the paradox of mentorship between emotional safety and independent growth

Although formal mentorship is challenging to maintain, the mentors act as a central support figure, offering emotional and professional guidance. They emphasize availability, visibility, and psychological safety.

I’m there for them; visible, present, and available. (R5)

This illustrates a form of relational anchoring that helps counterbalance the insecurity and vulnerability often experienced by new employees entering complex clinical environments.

The quote underscores the emotional dimension of mentorship, in which the mentor’s presence serves not only a pedagogical function but also a protective, relational one. Feeling seen, supported, and connected is foundational for newly graduated nurses who must navigate high expectations while still forming their professional identity. Isolation, in contrast, can impede learning, reduce confidence, and contribute to early discouragement:

They must never feel alone. That is the worst feeling they can have (R7)

Thus, emotional availability becomes a critical condition for both learning processes and long-term retention, ensuring that newly graduated nurses must never feel alone.

At the same time, mentoring is described as a paradoxical practice one that requires being “present” without becoming intrusive. The mentor must strike a balance between offering guidance and creating space for independent growth, allowing novices to experiment, make decisions, and develop their own clinical judgment. This balance is reflected in the statement: *“Give them time to reflect and think.” (R1)* Here, the emphasis on reflective space points to a deeper pedagogical principle: that meaningful learning requires time for sensemaking, not merely exposure to tasks.

Reflection time enables mentees to move beyond passive participation and toward active integration of experience. By allowing newcomers to process situations at their own pace, mentors foster autonomy, critical thinking, and internal motivation. This approach aligns with the idea that professional identity is not simply transmitted; it is co-constructed through guided practice, relational support, and opportunities for self-directed interpretation.

The paradox of mentorship being accessible yet restrained, therefore becomes a central dynamic in the onboarding process. Effective mentors provide a safety net that supports confidence and emotional security, while simultaneously withdrawing enough to allow independent mastery to emerge. Through this subtle oscillation between presence and invisibility, mentors contribute to both immediate well-being and the long-term development of competent, reflective practitioners.

### Oscillating between standardized competence and evolving professional identity

There is an indication that the structured program supports quality of care, competence development, professional identity, and retention. The two-year framework allows nurses to immerse themselves in the specialty and build confidence.

It’s a huge competence boost - for the nurses and for the patients. (R8)

This reflects the perceived impact of the structured two-year program on both individual and organizational levels. It suggests that the program supports a gradual, coherent professional development trajectory in which nurses gain confidence, clinical competence, and a clearer understanding of their professional role. Through sustained mentorship, guided reflection, and exposure to varied clinical situations, newly graduated nurses are not only able to deliver safer and more consistent patient care but also develop a stronger sense of mastery and autonomy in their practice.

At the organizational level, such structured programs contribute to building a more stable and competent workforce. As nurses progress through a predictable and supportive developmental pathway, they are more likely to feel valued, grounded, and connected to the team. This can enhance retention, particularly in specialties where complexity and workload can otherwise lead to early attrition. Additionally, the shared learning journey fosters a sense of collective identity and strengthens team culture, as participants develop common reference points, professional language, and mutual expectations. In sum, the program appears to function as both a professional safety net for individuals and a strategic investment in organizational sustainability.

I’m convinced we get better nurses out of it. (R2)

This quote indicates newly graduated nurses who are supported in engaging with updated practices through reflection, mentorship, and emotionally safe environments often grow into ambassadors for change. This is further elaborated:

They [newly graduated nurses] become ambassadors for updated practices (R6)

Hereby, the introduction programme contributes to improved quality of care, strengthens clinical competence, and enhances retention, as these nurses help foster inclusive, forward-thinking workplaces that encourage professional growth and long-term commitment.

When novices are introduced to evidence-based methods through structured reflection, mentoring relationships, and emotionally safe learning spaces, they are more likely to internalize these practices and carry them forward in their daily work. Over time, this exposure cultivates a sense of ownership and professional confidence that enables them not only to adopt updating practices but also to actively advocate for them. Such nurses can serve as catalysts for cultural renewal in clinical settings, encouraging colleagues to reflect on routines, challenge outdated habits, and engage with new knowledge.

Moreover, when early-career nurses feel empowered to contribute to change processes, it strengthens their identity as competent and valued professionals. This sense of meaning and influence supports both job satisfaction and retention, as individuals who experience themselves as part of a progressive and learning-oriented workplace are more inclined to remain in the organization. In this way, the development of “ambassadors” becomes mutually beneficial: it enhances quality of care through the diffusion of updated knowledge, and it contributes to a more inclusive, forward-thinking work environment that fosters long-term commitment and continual professional growth.

## Discussion

The findings of this study illuminate the decisive role that structured onboarding programs, grounded in competent leadership and meaningful mentor relationships, play in supporting newly graduated nurses’ transition into professional practice. The findings indicate that head nurses and mentors perceive onboarding not merely as a technical or administrative procedure, but as a multidimensional developmental process that integrates clinical, emotional, and relational dimensions. Newly graduated nurses enter complex and high‑demand environments where uncertainty, role ambiguity, and emotional strain are common. This reflects what has been reported in prior studies, which points to the following. A structured onboarding framework provides the necessary scaffolding to navigate these conditions, while the relational support from mentors enhances psychological safety and facilitates early professional identity formation [4, 20, 33].

Relational commitment is a foundational to early professional development, this is one of the clearest insights emerging from the findings concerns the profound emotional and professional investment mentors express in the success of newly graduated nurses. This relational orientation extends beyond formal mentorship tasks to include an authentic commitment to the well-being, development, and confidence of the novice nurse. This aligns with research emphasizing that mentorship in nursing is fundamentally relational: it involves empathy, attunement, shared professional values, and the cultivation of trust [34]. Such relationships create a sense of being seen, valued, and supported, which is essential for psychological safety and resilience during the transition into practice.

For newly graduated nurses, often navigating stress, uncertainty, and fear of inadequacy emotional support from mentors can mitigate isolation, enhance learning, and reduce anxiety [3, 33]. This emotional grounding is not peripheral to professional development; it constitutes a form of affective learning that shapes confidence, identity, and motivation. The mentors’ relational commitment therefore functions as a stabilizing force, enabling new graduates to engage more fully in clinical learning and reflection [2].

The second major theme, “Structured Planning and Individualized Support,” highlights the core tension but also synergy between standardization and personalization in onboarding. Structured onboarding offers clarity, predictable progression, and well-defined competencies, ensuring alignment with organizational and patient safety expectations. At the same time, the findings reveal that mentors continuously adapt their pedagogical approach, considering the unique learning histories, prior clinical experience, and personal characteristics of newly graduated nurses, this corresponds with existing literature [13, 18].

This dual architecture resonates strongly with adult learning theory, which underscores that adults learn best when instruction is relevant, placed appropriately, and responsive to their individual needs [13, 34]. The findings suggest that individualized support within a structured program is not simply beneficial but necessary: it fosters engagement, strengthens motivation, and accelerates competent development. A one‑size‑fits‑all approach risks overlooking the diverse trajectories new graduates bring with them; tailored mentorship, by contrast, enhances both learning and belonging.

The experiences described by mentors and leaders align closely with Bridges’ transition theory, which considers transition not as a single event but as a psychological process involving phases of “ending,” “neutral zone,” and “new beginning” [35] Newly graduated nurses appear to move through these phases more effectively when supported by both the structure, clear expectations and competencies, and the relational dimension (emotional support and guidance) of onboarding.

Similarly, Tønnesvang’s theory of vitalizing psychology contributes a valuable lens. According to Tønnesvang, environments that meet individuals’ needs for autonomy, competence, relatedness, and meaning enable psychological “oxygenation” that is, they energize development, identity formation, and engagement [36]. Structured onboarding, especially when anchored in relational commitment, clearly supports these conditions. Competence is developed through structured learning, relatedness through mentorship, autonomy through increased responsibility over time, and meaning through integration into professional culture and patient care.

A critical implication of these findings concerns workforce retention, which remains a pressing challenge in healthcare systems experiencing persistent nursing shortages. This is in line with earlier studies, that describe newly graduated nurses who encounter emotional security, structured support, and professional affirmation early in their careers are significantly more likely to remain in their positions [13, 37]. Retention is thus influenced not only by job satisfaction but by whether new nurses experience themselves as competent, valued contributors to organizational goals. Structured onboarding thereby supports both professional development and organizational resilience. By reducing turnover, organizations safeguard continuity of care, minimize recruitment costs, and maintain a stable, experienced workforce.

The study also underscores a strong connection between structured onboarding, competence development, and patient safety. When newly graduated nurses are supported through meaningful mentorship and clear expectations, they gain confidence more rapidly, make safer clinical decisions, and are better able to identify and communicate concerns [33]. This is congruent with earlier empirical findings that show that contributes to a culture where safety, reflection, and collaboration are embedded values rather than aspirational ideals [11, 38].

Leaders play a crucial role in shaping this culture. By prioritizing mentorship, fostering psychological safety, and investing in structured development pathways, they cultivate organizational environments where reflective practice and continuous improvement are natural extensions of daily work.

### Study limitations

The study is limited by its exclusive focus on mentor and leader perspectives. Future research should incorporate longitudinal designs and long-term effects on professional development, retention, and workplace integration [13, 39].

By employing Ricoeur’s three levels of interpretation and adhering to ethical considerations in line with the Helsinki Declaration, this study aims to provide a thorough and nuanced understanding of the significance of structured introduction programs for newly graduated nurses from the perspective of mentors and leaders.

### Future research / Implications

While this study provides valuable insights into the perceptions of nurse leaders and mentors regarding structured onboarding programs, further research is needed to explore the long-term effects of these programs on professional development and retention. Longitudinal studies that track the progress of newly graduated nurses over time could provide deeper insights into the lasting impact of mentorship and structured support on their careers.

Furthermore, future research may extend our earlier investigations by undertaking an in-depth examination of newly graduated nurses’ own experiences, thereby advancing a more nuanced and comprehensive understanding of their needs and expectations during the transition into professional practice.

Exploring the perspectives of diverse nursing populations, including those from underrepresented backgrounds, could also enhance the inclusivity and effectiveness of onboarding programs [34].

Furthermore, investigating the role of technology in facilitating structured onboarding and mentorship could yield valuable insights. As healthcare continues to evolve, understanding how digital tools can support the onboarding process may enhance the accessibility and effectiveness of mentorship programs. Tracking systems may support clinical leaders in supporting mentorship activities; however, their use should be carefully balanced to ensure that they complement rather than diminish the relational anchoring and emotional presence that participants highlighted as essential to effective mentorship.

Finally, the study points to the need for further research into which specific components of onboarding such as mentor training, simulation-based learning, peer support, or digital and hybrid formats most effectively enhance competence, well-being, and retention. Understanding how structured onboarding interacts with local organizational cultures will also be essential for refining models that support sustainable professional growth.

## Conclusion

This study demonstrates that a structured introduction program grounded in committed leadership and relational mentoring plays a pivotal role in supporting the transition from nursing student to professional nurse. The findings indicate that such programs create a safe and supportive learning environment, strengthen professional identity and clinical competence, and foster confidence among newly graduated nurses. Moreover, effective onboarding requires substantial organizational and emotional investment, highlighting the importance of clear structure, adequate resources, and sustained communication.

From both mentor and leader perspectives, onboarding emerges not merely as an administrative process but as a relational and developmental practice that shapes team culture, professional growth, and care quality. Despite practical constraints, the strong commitment to individualized mentoring underscores the transformative potential of structured introduction programs. These programs contribute to workforce retention, enhance patient care, and support the development of a competent, confident, and resilient nursing workforce.

Overall, the study underscores that personal commitment and organizational support are mutually reinforcing elements. Investing in comprehensive onboarding is therefore essential—not only for ensuring a successful transition for newly graduated nurses but also for strengthening the sustainability and quality of nursing practice.

## Supplementary Information

Below is the link to the electronic supplementary material.

Supplementary Material 1

## Data Availability

The datasets generated and analyzed during the current study are available from the corresponding author upon reasonable and academically relevant requests.

## Declarations

### Declaration

This manuscript reports a qualitative research study examining mentors’ and leaders’ experiences with a structured onboarding program for newly graduated nurses. No large language model (LLM) was used.
