# Supplementary material for: From orientation to integration: organizational leadership and mentorship for newly graduated nurses – a qualitative hermeneutic-phenomenological study
Source: BMC Nurs. 2026 Jun 15;25:744. doi: 10.1186/s12912-026-04886-4 (PMC13495472; doi:10.1186/s12912-026-04886-4)
Supplement: Supplementary file 1 [file 12912_2026_4886_MOESM1_ESM.docx]

**Interview Guide for leaders and mentors**

Interview questions for the project on introduction programme

We would like to talk with you about your experiences working with newly graduated nurses participating in the introduction programme. The interview will be audio‑recorded, and all information will be anonymized in the publication of our study results.

- Can you tell me about your experience as a leader or mentor and your time working in this department?
- How would you describe your role as a leader or mentor in relation to newly graduated nurses in the introduction programme?
- What kinds of competencies, experiences, or qualifications do you feel support you in carrying out the mentor role?
- What opportunities do you see in the introduction programme?
- Can you share what you feel has worked well, as well as any challenges you have encountered?
- Looking back at the start-up and implementation of the introduction programme, what do you think you or your department have learned?
- How do you understand the significance of the introduction programme for newly graduated nurses?
- How would you describe the impact the introduction programme has had on the workplace community in your department?
- In your view, how has the introduction programme influenced the quality of nursing in your department?
- Can you tell me about what you do to help ensure that the mentor is able to follow and support the newly graduated nurse?
- What do you think is needed to further strengthen or improve the programme going forward?
- When you encounter challenges in your role as a leader or mentor, where do you usually turn for support?
- Is there anything else you would like to share that we have not talked about yet?

Many thanks for your time and participation
